# Detection of left atrial appendage thrombus by dual-energy computed tomography-derived imaging biomarkers in patients with atrial fibrillation

**DOI:** 10.3389/fcvm.2022.809688

**Published:** 2022-07-22

**Authors:** Wenhuan Li, Mingxi Liu, Fangfang Yu, Weiwei Zhu, Xianbo Yu, Xiaojuan Guo, Qi Yang

**Affiliations:** ^1^Department of Radiology, Beijing Chao-Yang Hospital, Capital Medical University, Beijing, China; ^2^Department of Echocardiography, Heart Center, Capital Medical University, Beijing, China; ^3^CT Collaboration, Siemens Healthineers Ltd., Beijing, China

**Keywords:** left atrial appendage thrombus, left atrial thrombus, computed tomography, dual-energy CT (DECT), atrial fibrillation (AF)

## Abstract

**Aims:**

This study aimed to assess the diagnostic performances of dual-energy computed tomography (CT)-derived iodine concentration and effective atomic number (Z_*eff*_) in early-phase cardiac CT in detecting left atrial appendage (LAA) thrombus and differentiating thrombus from spontaneous echo contrast (SEC) in patients with atrial fibrillation using transesophageal echocardiography (TEE) as the reference standard.

**Methods and results:**

A total of 389 patients with atrial fibrillation were prospectively recruited. All patients underwent a single-phase cardiac dual-energy CT scan using a third-generation dual-source CT. The iodine concentration, Z_*eff*_, and conventional Hounsfield units (HU) in the LAA were measured and normalized to the ascending aorta (AA) of the same slice to calculate the LAA/AA ratio. Of the 389 patients, TEE showed thrombus in 15 (3.9%), SEC in 33 (8.5%), and no abnormality in 341 (87.7%) patients. Using TEE findings as the reference standard, the respective sensitivity, specificity, positive predictive value, and negative predictive value of the LAA/AA HU ratio for detecting LAA thrombus were 100.0, 96.8, 55.6, and 100.0%; those of the LAA/AA iodine concentration ratio were 100.0, 99.2, 83.3, and 100.0%; and those of the LAA/AA Z_*eff*_ ratio were 100.0, 98.9, 79.0, and 100.0%. The areas under the receiver operator characteristic curve (AUC) of the LAA/AA iodine concentration ratio (0.978; 95% CI 0.945–1.000) and Z_*eff*_ ratio (0.962; 95% CI 0.913–1.000) were significantly larger than that of the LAA/AA HU ratio (0.828; 95% CI 0.714–0.942) in differentiating the thrombus from the SEC (both *P* < 0.05). Although the AUC of the LAA/AA iodine concentration ratio was larger than that of the LAA/AA Z_*eff*_ ratio, no significant difference was found between them (*P* = *0.259*).

**Conclusion:**

The dual-energy CT-derived iodine concentration and the Z_*eff*_ showed better diagnostic performance than the conventional HU in early-phase cardiac CT in detecting LAA thrombus and differentiating the thrombus from the circulatory stasis. However, these results need to be validated in large-cohort studies with late-phase images.

## Introduction

Left atrial (LA) thrombus is common in patients with atrial fibrillation, with a prevalence of 13–15%, and most frequently occurs in the left atrial appendage (LAA) (1–3). The presence of thrombus in LA or LAA is considered to be a contraindication to electrical or pharmacological cardioversion or catheter ablation in patients with atrial fibrillation (4). Transesophageal echocardiography (TEE) is the gold standard in detecting or excluding LA or LAA thrombus before catheter ablation (5, 6). However, this technique is semi-invasive and carries physical discomfort for patients (7). Significantly, during the coronavirus disease 2019 pandemic, the performance of TEE carries a heightened risk for transmission of this viral infection, especially in non-intubated patients, secondary to the aerosolization of a large amount of virus due to coughing or gagging (7, 8). Therefore, it is important to develop a valid imaging modality alternative to TEE to exclude LAA.

Computed tomography (CT) is routinely performed before catheter ablation, as it provides the exact anatomical details of the LA and pulmonary veins (9–11). Indeed, a uniphasic standard CT cannot discriminate thrombus from flow stasis or stagnation when an iodine contrast-filling defect is present in LAA, owing to the lack of organized LA and LAA contraction in patients with atrial fibrillation (10, 12). The term spontaneous echo contrast (SEC) was coined to refer to this flow stasis visualized by echocardiographic examination (8). To overcome the issue of delayed filling due to stasis, a two-phase scan protocol was proposed, in which a second set of images is acquired following a short time delay after the initial scan (13). A meta-analysis showed that when delayed imaging was performed by this two-phase scan protocol, the specificity increased from 92 to 99% and the positive predictive value (PPV) increased from 41 to 92% (14). Likewise, a double-contrast single-phase scan has shown promising results despite a double-contrast load (15, 16). But, the two-phase scan protocol and double-contrast single-phase scan protocol are only applicable for some LAA stasis, owing to their limited ability to discern tissue characteristics.

The recently introduced dual-energy CT-derived iodine concentration can differentiate iodine from other materials through the material decomposition method. This is valuable for differentiating non-enhancing thrombus from other iodine-enhancing phenomena, such as SEC. Nevertheless, there exists an absolute measurement error in iodine quantification, especially when measured at lower iodine concentrations, such as severe SEC or patients with large-body type (17–19), which weaken the ability to distinguish thrombus. Another dual-energy CT-derived imaging biomarker called effective atomic number (Z_*eff*_) can chemically qualify the type of material. Previous studies have shown the potential of this technique in pilot populations enrolled for various disorders (20–24). Nevertheless, its abilities to detect LAA thrombus and differentiate LAA thrombus from circulatory stasis are unclear.

Thus, this study aimed to assess the diagnostic performances of dual-energy CT-derived iodine concentration and Z_*eff*_ in detecting LAA thrombus and differentiating thrombus from SEC in patients with atrial fibrillation using TEE as the reference standard.

## Materials and methods

### Patient selection

**FIGURE 1:**
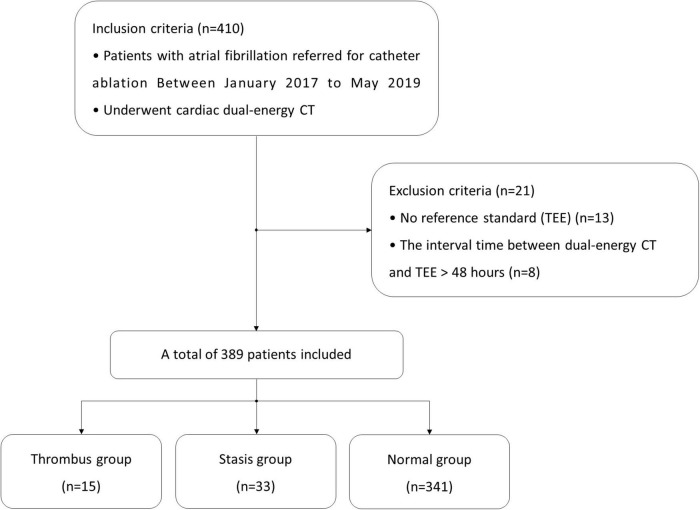
Flowchart for patient enrollment.

From January 2017 to May 2019, we prospectively enrolled consecutive patients with atrial fibrillation referred for catheter ablation. Figure 1 outlines the study protocol. All patients underwent TEE for the exclusion of LAA thrombi before undergoing catheter ablation and underwent cardiac dual-energy CT for anatomical assessment of the pulmonary veins, LA, and LAA. The interval time between dual-energy CT and TEE was less than 48 h. This study was approved by the institutional ethics committee, and written informed consent was obtained from each patient.

### Dual-energy cardiac computed tomography examination

Computed tomography was performed by a third-generation dual-source CT (SOMATOM Force; Siemens Healthineers, Forchheim, Germany) in dual-energy mode prospective ECG-gating technique. The CT scan parameters were as follows: 2 mm × 64 mm × 0.6 mm acquisition collimation with z-flying focal spot technique. An automated tube current modulation (Care Dose 4D, Siemens Healthcare) was used in scanning. One tube of the dual-source CT system was operated with 127 reference mAs per rotation at Sn 150 kV, and the second tube was automatically operated with 444 reference mAs per rotation at 70 kV. All CT scans were performed in cranio-caudal direction at a supine position during a mid-inspiratory breath-hold.

Contrast media were injected by a dual-syringe injector (Stellant D, Medrad, Indianola, MS, United States) using an 18-gauge intravenous needle placed in the right antecubital vein. A triphasic injection protocol was used. Firstly, 40 mL of contrast agent (Iopromide, Ultravist 370, 370 mg/ml, Bayer-Schering Pharma, Berlin, Germany) was administered. Thereafter, there was a 15-s delay before administration of the second bolus of contrast agent (40 ml). Finally, 30 ml of saline was administered. The injection rates were 4 ml/s for all phases. Contrast agent application was controlled by a bolus tracking technique. After the second bolus injection, there was a 5-s delay before the monitoring scan. A region of interest was placed in LA, and image acquisition was automatically started 7 s after the attenuation reached the predefined threshold of 100 HU.

### Dual-energy cardiac computed tomography image post-processing and evaluation

A dual-energy CT post-processing was performed by a dedicated clinical radiology workstation (Syngo Via, VB10, Siemens Healthcare). The acquired 70 kV and Sn150 kV images were transferred to the workstation and loaded into the dual-energy application software. This software allows the automated derivation of iodine maps and effective atomic number maps and obtains quantitative iodine concentration (in mg/ml) and effective atomic number values within a user-designated region of interest (ROI).

All CT images were assessed by two experienced readers who were blinded to echocardiographic and clinical information. Disagreements between readers were settled by consensus. If the entire LAA was not fully opacified with contrast media by visual assessment, a filling defect was deemed to be present. We then placed an ROI inside the filling defect in the LAA to determine the iodine concentration and Z_*eff*_. To account for hemodynamic inter-patient variations, all the measured parameter values in the ROI of the lesion were normalized to the ascending aorta (AA) in the same axial plane.

### Transesophageal echocardiography examination

A TEE was performed using commercially available echocardiograms (Toshiba Nemio, Tokyo, Japan) with a 5.0-MHz multiplane transducer. With the patient lying in the left lateral decubitus position and monitored by means of a three-lead electrocardiogram, the TEE probe was introduced into the esophagus with the transducer facing anteriorly (25). The LAA was imaged by placing the probe at the level of the mid-esophagus including 0, 45, 90, and 135 angled views. A thrombus was defined as an “echo-dense mass” that was distinct from the adjacent normal tissue without any detectable Doppler flow velocity and signs of vascularization on color doppler in the LAA. SEC was defined as hyperechogenic sparkling that could not be eliminated by adjusting the gain settings in the presence of residual flow velocity in the LAA (10). SEC was not regarded as a thrombus. The severity of SEC was classified according to the following criteria: grade 0, no echogenicity was observed (normal group); grade 1 (mild), minimal echogenicity, imperceptible at “normal” gain settings, may be detectable only transiently during the cardiac cycle, located in the left atrial appendage, or sparsely distributed in the left atrial cavity; grade 2 (mild to moderate), more dense swirling pattern, detectable without increased gain settings, with a similar distribution to mild; grade 3 (moderate), dense swirling pattern in the left atrial appendage, generally associated with somewhat lesser intensity throughout the main left atrial cavity, and may fluctuate in intensity but is detectable constantly throughout the cardiac cycle; and grade 4 (severe), intense echodensity and very slow swirling patterns in the left atrial appendage, usually with similar density in the main cavity (26).

### Statistical analysis

All statistical analyses were performed using SAS version 9.4 (SAS Institute Inc., Cary, NC, United States) or MedCac version 11.4.1 (Medcalc Software, Mariakerke, Belgium). Continuous variables were expressed as mean ± SD, and categorical variables were expressed as frequencies or percentages. Student’s *t*-test and the Wilcoxon test were performed for normally and non-normally distributed variables, respectively.

Using TEE as the reference standard for the diagnosis of a thrombus formation, the sensitivity, specificity, PPV, negative predictive value (NPV), and accuracy of iodine quantification and Z_*eff*_ for the detection of LAA thrombi were calculated. Additionally, using TEE as the reference standard for the diagnosis of a thrombus formation, the diagnostic performance was quantified using receiver operating characteristic (ROC) analysis, and the areas under the curves (AUCs) were compared using the DeLong method. A two-tailed *p*-value < 0.05 was considered to be statistically significant. Intraclass correlation coefficients were used to assess intraobserver and interobserver agreements in the measurements of iodine concentration and Z_*eff*_ in 10 randomly selected patients.

## Results

The characteristics of the study population are summarized in Table 1. A total of 389 patients (219 men and 170 women, 61.7 ± 8.2 years) were enrolled and analyzed. In all cases, the interval time between dual-energy CT and TEE was less than 48 h and within 24 h in 80% of the cases. The image quality of all dual-energy CT and TEE examinations was considered acceptable for evaluation. The mean estimated radiation effective dose was 2.2 ± 0.4 mSv (dose–length product × 0.014 mSv/mGycm).

**TABLE 1:** Characteristics of the study population (*n* = 389).

Characteristics	Value
Age (years) [mean ± SD]	61.7 ± 8.2
Sex [male/female]	219/170
BMI [kg/m^2^; mean ± SD]	25.3 ± 3.9
CHA2DS2-VASc score [mean ± SD]	1.4 ± 1.1
Hypertension (%)	120 (31%)
Hypercholesterolemia (%)	85 (22%)
Diabetes mellitus (%)	89 (23%)
Current or prior cigarette smoking (%)	97 (25%)
Warfarin	46 (12%)
NOAC	311 (80%)

Values are n (%).

SD, standard deviations; BMI, body mass index; NOAC, novel oral anticoagulants.

Of the 389 enrolled patients, TEE demonstrated thrombus in 15 (3.9%), SEC in 33 (8.5%), and no abnormality in 341 (87.7%) patients. The resultant severity of SEC was categorized as severe in 4 patients, moderate in four patients, mild to moderate in 10 patients, and mild in 15 patients on TEE. All thrombi were located in the LAA. On dual-energy CT, the mean LAA/AA iodine concentration ratio was −0.23 ± 0.14 for the thrombus group, 0.09 ± 0.13 for the SEC group, and 0.80 ± 0.29 for the normal group (no thrombus or SEC). The mean LAA/AA Z_*eff*_ ratio was 0.53 ± 0.07 for the thrombus group, 0.69 ± 0.11 for the SEC group, and 0.98 ± 0.19 for the normal group. The mean LAA/AA Z_*eff*_ ratio and the LAA/AA iodine concentration ratio were significantly different among the three groups (*p* < 0.001).

Using TEE findings as the reference standard, the sensitivity, specificity, PPV, and NPV of the LAA/AA HU ratio were 100.0, 96.8, 55.6, and 100.0%, respectively, in detecting the LAA thrombus (Table 2) by using the LAA/AA iodine concentration ratio, were 100.0, 99.2, 83.3, and 100.0%; and by using the LAA/AA Z_*eff*_ ratio, were 100.0, 98.9, 79.0 and 100.0%, respectively (Table 2).

**TABLE 2:** Statistical results for the detection of left atrial appendage (LAA) thrombus (*n* = 389).

Image series	Accuracy	Sensitivity	Specificity	PPV	NPV
LAA/AA iodine concentration ratio	99.2 (386/389) [97.8–99.8]	100.0 (15/15) [78.2–100]	99.2 (371/374) [97.7–99.8]	83.3 (15/18) [58.6–96.4]	100.0 (371/371) [99.0–100]
LAA/AA Z_*eff*_ ratio	99.0 (385/389) [97.4–99.7]	100.0 (15/15) [78.2–100]	98.9 (370/374) [97.3–99.7]	79.0 (15/19) [54.4–94.0]	100.0 (370/370) [99.0–100]
LAA/AA HU ratio	96.9 (377/389) [94.7–98.4]	100.0 (15/15) [78.2–100.0]	96.8 (362/374) [94.5–98.3]	55.6 (15/27) [35.3–74.5]	100.0 (362/362) [99.0–100]

Values are% (raw data) [95% CI].

PPV, positive predictive value; NPV, negative predictive value.

**FIGURE 2:**
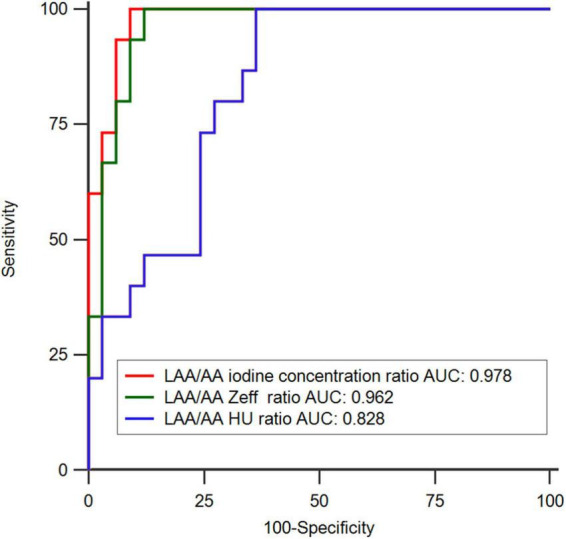
Receiver operating-characteristic curve (ROC) analysis in differentiating thrombus from circulatory stasis using transesophageal echocardiography (TEE) as the reference standard. The areas under the ROC (AUC) of LAA/AA iodine concentration ratio (0.978; 0.945–1.000) and LAA/AA Z_*eff*_ ratio (0.962; 0.913–1.000) showed significantly larger than that of LAA/AA HU ratio (0.828; 0.714–0.942) (*P* < 0.05).

**FIGURE 3:**
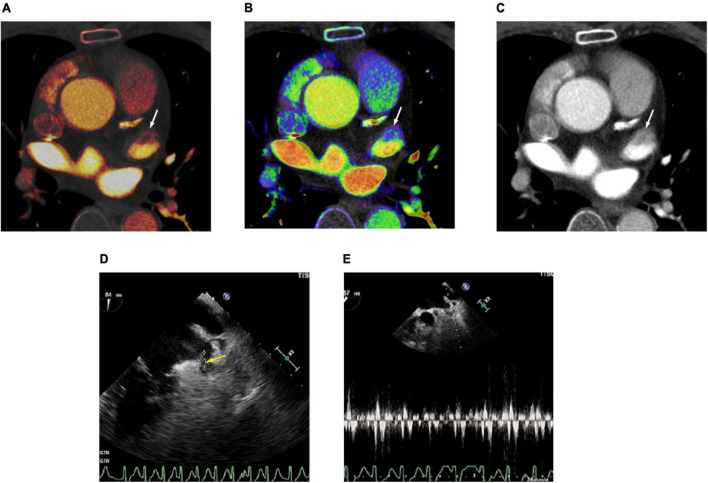
Cardiac computed tomography (CT) and transesophageal echocardiography (TEE) images in 78-year-old man with left atrial appendage (LAA) thrombus and spontaneous echo contrast (SEC). **(A)** Dual-energy CT-derived iodine map shows filling defect in LAA (arrow), with LAA/AA iodine concentration ratio of –0.18 (true positive finding). **(B)** Dual-energy CT-derived Z_*eff*_ image shows filling defect in LAA (arrow), with LAA/AA Z_*eff*_ ratio of 0.54 (true positive finding). **(C)** Conventional CT image shows filling defect in the LAA (arrow) with LAA/AA HU ratio of 0.11 (true positive finding). **(D)** TEE image demonstrates thrombus (arrow) and SEC in LAA. **(E)** Doppler flow measurement of TEE shows reduced emptying velocity of 38 cm/s in LAA besides the thrombus. For quantitative assessment, LAA/AA iodine concentration ratio ≤ –0.09, LAA/AA Z_*eff*_ ratio of ≤0.61 and LAA/AA HU ratio of ≤0.33 was considered positive for thrombus.

**FIGURE 4:**
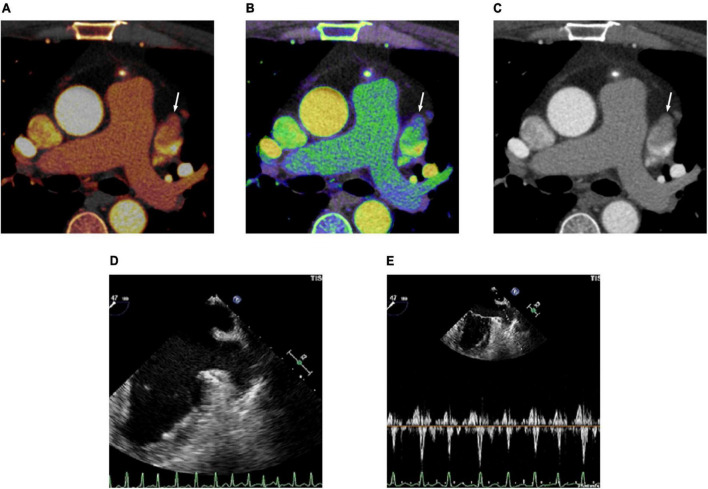
Cardiac computed tomography (CT) and transesophageal echocardiography (TEE) images in 48-year-old man with spontaneous echo contrast (SEC). **(A)** Dual-energy CT-derived iodine map shows filling defect in the left atrial appendage (LAA) (arrow) with LAA/AA iodine concentration ratio of 0.13 (true negative finding). **(B)** Dual-energy CT-derived Z_*eff*_ image shows filling defect in the LAA (arrow) with LAA/AA Z_*eff*_ ratio of 0.68 (true negative finding). **(C)** Conventional CT image shows filling defect (arrow) in the LAA with LAA/AA HU ratio of 0.24 (false positive finding). **(D)** TEE image demonstrates SEC in LAA. **(E)** Doppler flow measurement of TEE shows reduced emptying velocity of 37 cm/s but measurable flow throughout the LAA.

In the ROC analysis, the AUCs of the LAA/AA iodine concentration ratio (AUC 0.978; 95% CI 0.945–1.000) and the LAA/AA Z_*eff*_ ratio (0.962; 0.913–1.000) were significantly larger than that of the LAA/AA HU ratio (0.828; 0.714–0.942) in differentiating thrombus from SEC (both *p* < 0.05; Figure 2). Although the AUC of the LAA/AA iodine concentration ratio was larger than that of the LAA/AA Z_*eff*_ ratio (0.978 vs 0.962), no significant difference was found between them (*p* = 0.259). The best cutoff values for detecting an LAA thrombus and differentiating thrombus from SEC were -0.09 for the LAA/AA iodine concentration ratio, 0.61 for the LAA/AA Z_*eff*_ ratio, and 0.33 for the LAA/AA HU ratio. Figure 3 shows representative dual-energy CT images from a patient with LAA thrombus and SEC, and Figure 4 shows representative dual-energy CT images from a patient with LAA SEC.

Intraobserver and interobserver agreements for the measurement of the LAA/AA iodine concentration ratio (ICC = 0.88, ICC = 0.81) and LAA/AA Z_*eff*_ ratio (ICC = 0.86, ICC = 0.82) were all excellent.

## Discussion

This study was conducted to assess the diagnostic performances of dual-energy CT-derived iodine concentration and Z_*eff*_ in detecting LAA thrombus and differentiating thrombus from SEC in patients with atrial fibrillation using TEE as the reference standard. This study demonstrated that the diagnostic performances of iodine concentration and Z_*eff*_ were superior to conventional HU in detecting LAA thrombus and differentiating thrombus from circulatory stasis. Although the diagnostic performance of iodine concentration measurements was better than that of Z_*eff*_ measurements, no significant difference was found between them.

Recent studies have shown that conventional CT has high sensitivity and NPV, but poor specificity and PPV in detecting LAA thrombus because CT has a limited capacity in differentiating thrombus from circulatory stasis when an LAA-filling defect is present (10, 12, 27). Kim et al. (28) reported that the PPV and NPV of conventional CT measurements (HU) for the detection of severe spontaneous echo contrast and thrombus were 31 and 99%, respectively. Budoff et al. (29) reported that the PPV and NPV of conventional CT for the detection of LAA thrombus were 51.6 and 100%, respectively. Compared with previous studies, the PPV of this study was slightly higher (55.6%), which may be due to the use of the double-contrast single-phase scan protocol. The double-contrast single-phase scan protocol is an alternative protocol to improve the ability to detect LAA thrombus, involving only one-phase CT scan after two separate injections of contrast agent without delayed CT scan. Teunissen et al. (30) reported a PPV of 7.7% when using two contrast boluses (30 and 70 ml) with 25-s inter-bolus delay. However, another study by Hur et al. (15) showed a PPV of 100% when using two contrast boluses (50 and 70 ml) with 180-s delayed acquisition after the test bolus (first bolus). This may indicate that the two contrast boluses combined with the optimal interval time between injections or optimal delayed acquisition time is of great significance in improving the ability to detect LAA thrombus.

Another CT protocol designed to improve the diagnostic ability to detect LAA thrombus is the two-phase scan protocol, in which the second set of images is acquired following a short time delay after the initial scan, recommended using a 60-s delay from contrast peak detected by bolus tracking (13). A recent meta-analysis showed that the specificity increased from 92 to 99% and the PPV increased from 41 to 92% by using the two-phase scan protocol with delayed imaging (14). Notably, for the two-phase scan protocol, an optimal acquisition time for the second scan is most crucial but difficult aspects because the circulation time depends on the individual involved. If the acquisition time is not properly chosen, the contrast medium will run out or will not be fully filled in LAA. Moreover, the double-contrast single-phase protocol and the two-phase scan protocol are only applicable for some LAA stasis, owing to their limited ability to discern the tissue characteristics.

Compared with conventional single-energy CT, dual-energy CT is not only a powerful technique to obtain material-specific information by adding a second set of data to the same scanned material but it also reduces beam-hardening artifacts (31, 32). Our study further enhanced the advantages of dual-energy CT. In our study, dual-energy CT-derived iodine quantification (AUC 0.978 ± 0.017 vs 0.828 ± 0.058, *p* < 0.05) and Z_*eff*_ (AUC 0.962 ± 0.025 vs 0.828 ± 0.058, *p* < 0.05) showed significantly higher diagnostic ability than conventional HU measurements in the detection of LAA thrombus. Such observations are caused by using materials with different elemental compositions that can represent the same CT values (HU), making the differentiation and classification of different tissues extremely challenging. Moreover, the conventional HU measurements may be affected by confounding factors characterized by both the unenhanced CT attenuation densitometry and the contrast-enhanced densitometry. Conversely, the iodine quantification, unaffected by unenhanced CT attenuation densitometry, has great potential to differentiate non-enhancing thrombi from enhancing intracardiac blood pools. In line with current observation, our group reported that iodine concentration measurements based on dual-energy dual-source CT were superior to conventional HU measurements (33) in detecting LAA thrombus. Moreover, Hur et al. (34) have reported that iodine concentration measurements based on single-source dual-energy CT with a fast-kilovoltage-switching technique were better than conventional HU measurements in distinguishing thrombus from circulatory stasis.

Dual-energy CT-derived Zeff is a quantitative parameter that represents the mean atomic number of compounds or mixtures of various materials in tissue, rather than the absolute value of pure single chemical substance (35). Zeff describes the electronic changes at the atomic level. It can show subtle amount of iodine with higher sensitivity than CT attenuation in Hounsfield units (20, 21, 24). In our study, this electronic contribution from subtle amounts of iodine within a pseudo–filling defect provides useful information about the presence and accumulation of contrast material by chemically qualifying the focused type of material. Even if the manufacturer states that the Z_*eff*_ results for materials with a higher atomic number may not be correct, recent data are not in conflict with the manufacturer statement because the recent data are based on distinguishing the materials with different contents of higher atomic number and not based on the quantification of the absolute Z_*eff*_ value.

Z_*eff*_ measurements were compared with iodine measurements in our study. Interestingly, the diagnostic performance of iodine concentration measurements was better than that of Z_*eff*_ measurements in the detection of LAA thrombus, but without significant (AUC 0.978 vs 0.962, *p* > 0.05). Of 374 patients without thrombus, four were misdiagnosed with thrombus by Z_*eff*_ measurements and severe SEC were detected by TEE in these cases. The false-positive cases may be due to the iodine concentration being not measurable in the LAA at the time of data acquisition because contrast opacification might take longer in patients with atrial fibrillation who have very low filling and emptying velocities than in individuals in sinus rhythm. Likewise, these situations may also negatively affect the diagnostic performance of iodine concentration measurements in detecting LAA thrombus. Of 374 individuals without thrombus, three were misdiagnosed with thrombus by iodine concentration measurements and TEE showed severe SEC. However, undoubtedly, iodine concentration measurements and Z_*eff*_ measurements are more sensitive than conventional HU measurements in detecting the iodine presence and are relatively less affected by the scan time.

In this study, to minimize the variations of circulation status and scanning time in different patients, all the measured parameter values in the ROI of the lesion were normalized to the AA in the same axial plane. Additionally, to achieve a sufficient attenuation difference between iodine-enhancing and non-enhancing phenomena, a dual-enhanced protocol involving two injections of iodinated contrast media was followed, and a short delay time was used between administering the first and second contrast boluses.

Nowadays, although a few studies have evaluated the potential of dual-energy CT-derived Z_*eff*_ in pilot populations enrolled for various disorders (20–24), the ability for differentiation of LAA thrombus and circulatory stasis remains unclear. To the best of our knowledge, the application of Z_*eff*_ in detecting LAA thrombus has never been reported. The finding of this study can be considered a proof of concept of the feasibility of this novel technology in detecting LAA thrombus. Taken together, these study results suggest the potential of dual-energy CT as a promising non-invasive method for the identification of individuals with LAA thrombus.

## Limitations

This study has several limitations. First, this was a single-center analysis with a relatively small number of positive cases. Although small, the results are encouraging but require further studies with larger cohorts to validate. Second, TEE was used as the reference standard and the presence or absence of LAA thrombus was not confirmed by direct visual surgical specimens or inspection of the anatomy. Third, although the intervals between dual-energy CT and TEE were within 48 h, the risk of “new” thrombus formation would be low. We cannot entirely erase the possibility that this did occur between the two examinations. Fourth, we did not perform the two-phase CT scan protocol and the dual-energy CT-derived iodine concentration and Zeff were only compared with HU from early-phase CT instead of late-phase images. Future studies including the comparison with the two-phase scan protocol with late-phase images are required. Finally, the study was performed in a cohort with relatively low CHA2DS2-VASc score, which may limit generalizability.

## Conclusion

Dual-energy CT-derived iodine concentration and Z_*eff*_ showed better diagnostic performance than conventional HU in early-phase cardiac CT in detecting LAA thrombus and differentiating thrombus from circulatory stasis. However, these results need to be validated in large cohort studies with late-phase images.

## Data availability statement

The raw data supporting the conclusions of this article will be made available by the authors, without undue reservation.

## Ethics statement

The studies involving human participants were reviewed and approved by Institutional Review Board, Beijing Chao-Yang Hospital, Capital Medical University. The patients/participants provided their written informed consent to participate in this study. Written informed consent was obtained from the individual(s) for the publication of any potentially identifiable images or data included in this article.

## Author contributions

WL: writing–original draft preparation, conceptualization, methodology, and software. ML: data curation. FY: visualization and investigation. WZ: software and validation. XG and QY: project administration and supervision. All authors contributed to the article and approved the submitted version.

## Conflict of interest

XY was employed by CT Collaboration, Siemens Healthineers Ltd. The remaining authors declare that the research was conducted in the absence of any commercial or financial relationships that could be construed as a potential conflict of interest.

## Publisher’s note

All claims expressed in this article are solely those of the authors and do not necessarily represent those of their affiliated organizations, or those of the publisher, the editors and the reviewers. Any product that may be evaluated in this article, or claim that may be made by its manufacturer, is not guaranteed or endorsed by the publisher.

## Abbreviations

AUC: area under the receiver operating characteristic curve
CT: computed tomography
HU: Hounsfield units
Z_*eff*_: effective atomic number
TEE: transesophageal echocardiography
LAA: left atrial appendage
AA: ascending aorta
SEC: spontaneous echocardiographic contrast.
